# White Guinea yam (*Dioscorea rotundata* Poir.) landraces trait profiling and setting benchmark for breeding programs in the Republic of Benin

**DOI:** 10.1371/journal.pone.0273043

**Published:** 2022-08-17

**Authors:** Yêyinou Laura Estelle Loko, Charlemagne D. S. J. Gbemavo, Paterne A. Agre, Anicet G. Dassou, Octaviano Igor Yelome, Roger Idossou, S. Alban Etchiha Afoha, Eric Dadonougbo, Jeannette Fakorede, Alexandre A. Dansi

**Affiliations:** 1 Laboratory of Biotechnology, Genetic Resources and Plant and Animal Breeding (BIORAVE), National High School of Applied Biosciences and Biotechnologies (ENSBBA/UNSTIM), Dassa-Zoumé, Benin; 2 Laboratory of Applied Entomology (LEnA), National High School of Applied Biosciences and Biotechnologies (ENSBBA), National University of Sciences, Technologies, Engineering and Mathematics, Dassa, Benin; 3 International Institute of Tropical Agriculture (IITA), Ibadan, Nigeria; Shahjalal University of Science and Technology, BANGLADESH

## Abstract

To meet the high demand for white Guinea yam, there is a need to develop and release improved varieties to farmers. Unfortunately, low rate of adoption of most of the improved yam varieties by both producers and consumers was observed. Information regarding agronomic characteristics and food qualities of popular white Guinea yam landraces with high market value are not available to establish minimum standards to be considered by breeding programs. To fill this gap, surveys using rural appraisal tools were carried out in 20 villages and 16 markets throughout Benin. Data on the agronomic performance suggested that for an improved variety to be adopted by Beninese farmers it should have a minimum yield of 4.16 ± 0.15 kg per mound, and average number of marketable tubers of 1.23 ± 0.05, a mean tuber length of 36.41 ± 1.22 cm, and a minimum diameter of 25.44 ± 1.16 cm. The sensorial attributes for boiled and pounded tubers of this improved variety should have minimum score of 3.16 for texture, 0.75 for softness, 3.75 for elasticity, and 1.34 for colour during the sensory evaluation. The improved variety must also have a minimum average severity score of 1.1 for yam mosaic virus disease, 1.33 for anthracnose and 1 for nematodes. Landraces Amoula, Laboko, and Djilaadja should be considered as the standard for yield, sensory attributes, and tolerance to pest and diseases while landraces Danwari, Kodjewe, Mondji, and Gnidou should be characterized as possessing good flowering and fruit setting capacities for breeding programs.

## 1. Introduction

Yam (*Dioscorea* spp.) is a climbing crop species that produces edible underground tubers, which contribute to the food security and poverty alleviation in many developing countries [1]. About 94% of the global yam production is from West Africa with the Republic of Benin as the fourth world producer [2]. In this region, among the cultivated yam species, the white Guinea yam (*Dioscorea rotundata* Poir.) remains the most produced and preferred [3, 4]. Its tubers appear to be a good source of carbohydrates and essential minerals such as magnesium, zinc, iron and phosphorus [5]. White Guinea yam plays an important role in the socio-cultural life of local farming communities through festivals dedicated to the release of new yam tubers [6], and in the rural household economy [7].

Unfortunately, white Guinea yam production is constrained by numerous biotic and abiotic constraints resulting in yield reduction and varietal diversity loss [8]. According to Hounnou et al. [9], climate change could lead to significant reduction in Benin yam production up to 6.87% by 2025. To meet future demands for white Guinea yam and to ensure food security, there is a need to develop and release improved yam varieties for farmers [10]. However, the adoption rate of improved yam varieties introduced in the past in the Republic of Benin is low [11]. For example, the adoption rate of improved yam varieties (TDr 131 and TDr 205) introduced and popularized by the International Institute of Tropical Agriculture (IITA) in Benin was estimated at 37% in 2007 [12]. This low adoption rate could be due to several factors including poor match with consumer needs and preferences, poor dissemination, lack of seeds, but also under local conditions to the small adaptation of improved varieties over landraces [11, 13]. Although, improved yam varieties are known to have superior agronomic and food quality traits [14], unfortunately there is no defined minimum breeding standards for tuber yield, food quality, pest and disease tolerance to be considered when developing new white Guinea yam varieties.

Beninese farmers perceive popular and elite yam landraces as those having high yield potential, resistance or tolerance to many biotic and abiotic constraints [8, 15]. Therefore, it is important to determine the average agronomic performance across production zones of these popular yam landraces, which could serve as minimum breeding standards for acceptability of improved white Guinea yams by Beninese farmers. Presently, there is lack of information on the agronomic characteristics and food qualities of popular white Guinea yam landraces with high market value to allow the establishment of minimum quality standards for the national yam breeding program of Benin.

The selection of white Guinea yam landraces is usually based on a set of farmers’ preference criteria of which good yield, good quality and high market value are the most important [8]. Customer needs are now the focus of yam breeding programs in Africa [14]. Although efforts of several research programs have been devoted to white Guinea yam in the Republic of Benin, there is very limited information on the yield and market value of the popular landraces. However, this information is crucial to the development of new yam varieties with improved agronomic characteristics with a greater probability of adoption in the markets. To fill these gaps, it is important to explore the yam producing villages and local markets in order to identify the popular yam landraces and document their market value and agronomic performance including their on-farm phytosanitary status as white Guinea yam landraces are highly susceptible to pathogens [8, 16], which affect their yield and market values [17].

In addition to the good agronomic characteristics required in any improved yam variety, the sensory attributes of dishes based on this variety are major determinants of its adoption by both producers and consumers [18–20]. Indeed, the texture and taste of pounded and boiled yam, which are the most popular yam-based dishes in Benin Republic are the main sensory quality attributes of Beninese farmers, processors, and consumers [20, 21]. Therefore, it is important to determine the minimum standards of these sensory attributes of popular white Guinea yam in order to be locally adopted.

The objectives of this study were to: (i) identify the popular white Guinea yam landraces with high market value in the study area; (ii) assess the on-farm agronomic performance of these elite yam landraces across farmers’ fields and production zones; (iii) evaluate the sensory quality of the boiled and pounded yam of these elite yam landraces; (iv) establish the basic standards for yam breeding and selection programs in the Republic of Benin.

## 2. Materials and methods

### 2.1. Surveys

#### 2.1.1. Study area and site selection

The study was carried out in the intensive yam-growing areas of four regions (Collines, Donga, Atakora, and Borgou) located in the central (latitude 7° 45’ and 8° 40’ North and longitude 2° 20’ and 2° 35’ East) and northern (latitude 9° 00 ’and 12° 30’ North and longitude 1° and 30° 40’ East) regions of Benin. In Benin, yam is not produced in the far north because of drought stress and in the far south due to ignorance of cultural practices [8]. The rainfall pattern in central Benin straddles that of the bimodal distribution in the South and that of the unimodal distribution in the North. In the study areas, the rainfall varies between 1100 and 1200 mm/year and the temperature varies between 27 and 35°C. The vegetation is of the wooded savannah type in the Sudanese regions of the North and savannah in the Centre. The soils are predominantly tropical ferruginous concretion or ferrallitic.

Twenty villages were selected based on the yam production statistics collected from the agriculture and rural development office of each department. Sixteen yam local markets close to the chosen villages were also surveyed. Surveyed villages covered sixteen ethnic groups. These are Idaatcha, Tchabè, Nago, Mahi and Yoruba in central Benin; Yom, Ani, Lokpa, Natimba, Kotocoli, Gnindé, Ditamari, Wama and Berba in the north west; Bariba and Boko in the north east.

#### 2.1.2. Identification of popular yam landraces with high market value

Surveys were conducted using participatory research appraisal tools and techniques, such as group discussions and yam field observations [22]. In each village, a group of 20 yam producers was selected with the help of the chief of the village or head of farmers’ association for group discussions. After presentation of the research objectives to the farmers, they were asked to list the existing yam landraces in their village. The conservation status of each landrace was evaluated by using the foursquare analysis method [8, 23]. The foursquare analysis approach helped to classify existing landraces at the village level into four groups: landraces cultivated by many households on large areas (++); landraces cultivated by many households on small areas (+-); landraces cultivated by few households on large areas (-+), and landraces cultivated by few households on small areas (—). Through this method, the popular yam landraces (cultivated on large areas and by many households (++)) were then identified. A yam landrace was considered cultivated by many households when over 50% of the households of the village grew it; and on a large area when it was cultivated on more than 0.25 ha [24].

During focus group discussions, information on the agronomic, technological, and culinary performance of each yam landrace listed in each village were recorded. Fourteen variables (susceptibility to high soil moisture, tolerance to drought, susceptibility to poor soil, resistance to yam nematodes, resistance to yam mosaic virus (YMV), resistance to anthracnose, susceptibility to weeds, stay-green characteristic, relative productivity, storage aptitude of the fresh tuber, quality of chips, storage aptitude of the chips, quality of the pounded yam, and quality of the boiled yam) were used to assess the performance of each landrace. According to Loko et al. [15], yam landraces were scored 1 when it was unanimously recognized by the farmers as efficient (very good) and 0 when it was unanimously recognized by the farmers as inefficient.

Sixteen closest rural markets to the surveyed villages were selected for the estimation of the market value of the identified popular yam landraces. In each market, 5 to 12 yam wholesalers and retailers of yam were randomly selected for the survey. A total of 128 traders were surveyed in the study area. In the markets, yams were sold by retailers in small lots of 3 to 5 tubers per landrace, while the wholesalers sold them in lots of 50 to 100 tubers. It was observed in some markets such as Glazoué market that some traders were selling yam in kilograms using baskets and an adapted weighing scale. The market value of tubers of each yam landrace was estimated regarding the price per kg, the number of sellers and available quantity (in tons). For each yam landrace, five representative lots of tubers were selected and weighed to provide the peak price per kilogram for both wholesale and retail sales. The average price per kg of tubers of all popular yam landraces found in the markets was used as a standard to identify those considered to have high prices. A popular white Guinea yam landrace was considered as sold by many sellers when, Sc ≥ Ns/2 (Sc is the number of local yam variety sellers and Ns total number of randomly selected sellers in the market). A popular white Guinea yam landrace sold by many sellers with high price and available in large quantities (≥ 1 ton) were considered as having high market value.

### 2.2. On-farm agronomic performance of popular yam landraces with high market value

In each surveyed village, five yam producers were chosen for field visits with the help of the village chief and/or heads of farmer associations. These farmers were selected on the basis of similarity in their farming practices (monoculture, staking, mulching, and no use of fertilizers and pesticides), and in order to include all popular yam landraces with high market values identified at the village level. Three visits were made in each field at different yam development stages. During the first visit (yam germination stage), five plants of each identified popular and high market value landraces were randomly selected and tagged with white tape for data collection. During the second visit, at the vegetative yam development stage (10 to 12 weeks after planting), the vigour, flowering intensity and fruiting intensity were recorded according to the yam descriptors [25]. In addition, the presence/absence of yam mosaic virus (YMV) and anthracnose symptoms was assessed on the leaves using the standard descriptor for yam variety performance evaluation trials [26]. Disease symptom severity for anthracnose and YMV were scored using the scale 1 = no visible symptoms of disease; 2 = few symptoms of 1 to ~25% on the plant; 3 = symptoms covering ~26 to ~50% of the plant; 4 = symptoms on > 51% of the plant; 5 = severe necrosis and death of the plant [26]. At the last visit, at the yam harvest stage (6 to 8 months after planting depending on the landrace), the yield (kg per mound) and yield components (total number of tubers harvested per mound, weight, length and diameter of the longest tuber per mound) were evaluated. In addition, nematode (*Scutellonema bradys* Steiner and LeHew) damage on harvested yam tubers were assessed using the standard descriptor of Asfaw [26].

### 2.3. Sensory evaluation of popular yam landraces with high market value

Fresh matured tubers of each popular white Guinea yam landrace with high market value were collected from the surveyed villages. The yam tubers were washed with clean water to remove adhering soil and other undesirable materials in the laboratory of the Biotechnology, Genetic Resources and Animal and Plant Breeding (BIORAVE). The yam samples were sorted, hand-peeled using kitchen knives and then cut into slices of about 10 cm each. About 2 kg of yam tuber slices from each landrace were cooked (temperature of 100°C) with 200 ml of water. Cooking times were recorded as they varied from one yam landrace to the other. Qasa yam pounding machine (Model QYP-6000) was used for the pounding of the boiled yam samples for 3 min.

Four randomly selected boiled and pounded yam samples from each landrace were served to consumers to determine consumer acceptability and assess sensory characteristics. The yam dishes prepared from each landrace was coded. To determine the consumer acceptability of the dishes prepared from each landrace, 60 untrained consumer panellists from the students and staff members of the National High School of Applied Biosciences and Biotechnologies (ENSBBA) were selected as panellist based on their willingness to participate. A 5-level hedonic scale ranging from very unpleasant (1) to very pleasant (5) were used for the evaluation [20]. Subsequently, 15 panellists previously trained on the principles of scoring and assessment were recruited to assess the sensory characteristics (hardness, sweetness, colour, aroma, hardness and elasticity) of boiled and pounded tubers of each white Guinea yam landrace. A glass of water was provided to each panellist to rinse the mouth between two tasting sessions.

### 2.4. Ethics declarations

The research protocol was approved by the ethic committee of the National University of Sciences, Technologies, Engineering and Mathematics (UNSTIM). Interviews were carried out in accordance with the guidelines of the Declaration of Helsinki. Written informed consent was obtained from all participants prior to the interviews.

### 2.5. Data analysis

Descriptive (mean, percentage, covariance, etc.) and inferential statistics were used to analyse the data collected. The Hierarchical Cluster Analysis (HCA) was used to establish the relationship between the popular yam landraces with high market value and their agronomic and culinary characteristics as perceived by the surveyed farmers. The data analysis was performed using the R software [27] with the ade4 [28, 29], and cluster [30] packages. The dist. binary function was used to calculate Jaccard’s similarity and the *hclust* function was used to construct a dendrogram. Analysis of variance (ANOVA) was performed using the IBM SPSS software version 23. Before the ANOVA test, the data normality was tested using Levene’s test. To achieve normality and homogeneity of variances, yield and yield component data were log-transformed (log (x + 1)). Significant differences between means were separated using the Student Newman Keuls test (p < 0.05). Principal Component Analysis (PCA) was performed to describe the relationship between the popular yam landraces and their yield and yield components. Pearson correlation analysis was used to measure the degree of association between the total number of tubers harvested, yield in kg per mound, length, diameter and weight of marketable tubers per mound.

To classify and characterize the popular yam landraces with high market value, the data collected including agro-morphological and sensorial attributes (Table 1) were considered for analysis. The data were analysed using classification performed in two stages. First, dimension reduction was performed on the data matrix containing yam landraces in rows and groups of variables in columns using multiple factor analysis (MFA) to maximise variation between popular yam landraces in the study area. MFA is a factor analysis which builds not only on the variation between yam landraces, but also the influence of groups of variables on the variation [31]. Second, hierarchical cluster analysis (HCA) was performed on the output from the MFA with popular yam landraces grouped such that the within-group variability in landrace characteristic was minimized while between-group variability was maximized. Both MFA and HCA were performed in R software [32] using the Factoshiny package [33]. Finally, the average of each quantitative character in each of the resulting clusters were compared to the global mean for that characteristic using the v-test (Decision statistic) and Mann-Whitney test.

**Table 1:** Category and contributing variables for the multiple factor analysis for classifying popular yam landraces with high market value.

Category of variables	Variables	Unit or scoring
Yield	Weight of tubers	Kg
	Number of tubers	-
	Yield	kg/m^2^
	Height of the marketable tubers	cm
	Width of the marketable tubers	cm
Flowering	Cycle	1 or 2
	Sex	1 or 2
	Vigour	1 to 3
	Flowering	0 to 7
	Fructification	0 to 7
Resistance to yam viral infection	Incidence	%
	Severity	1 to 3
Resistance to anthracnose	Incidence	%
	Severity	1 to 3
Resistance to nematodes	Incidence	%
	Severity	1 to 3
Sensorial attributes	Colour	0 to 3
	Taste	0 to 3
	Aroma	%
	Texture	1 to 5
	Lump	%
	Elasticity	1 to 5

Disease incidence (*DI*) and severity index (*MS*) were calculated using the following formula [34]:

$$DI=\frac{\sum_{i=1}^{n}IP}{\sum_{i=1}^{n}PS}\times 100$$ (1)

with IP: Number of infested plants and PS: total number of plants.

$$MS=\frac{\sum_{i=1}^{n}S}{\sum_{i=1}^{n}IP}\times 100$$ (2)

with S score of infested plants and IP Infested plants.

## 3. Results

### 3.1. Identification of the popular white Guinea yam landraces with high market value

The number of white Guinea yam landraces identified per village varied from 2 to 16 with 8.7 landraces in average (Table 2). Subject to local names, 213 white Guinea yam landraces were inventoried throughout the surveyed villages with 116 landraces identified as popular at the village level. The great majority (62.1%) of the identified popular white Guinea yam landraces were found for sale in the markets surrounding the surveyed villages (Table 2).

**Table 2:** Popular white Guinea yam landraces identified per village and corresponding market.

Villages	Markets	NPLV	NPLM	Popular yam landraces
Adidokparou	Tchaourou	13	6	Déba, Yoroubadodoun*, Wonkaabou*, Yombini*, Tinonpeti, Saria*, Kokouman, Ahimon, Yonouan*, Baniouré, Laboko, Nonfonnan*, Nonnina*.
Akaradè	Bodi	12	9	Oroutani, Toubega, Morokorou, Lassirin, Ayaasso*, Danwari, Ossoukpana, Foutoukpêtê, Yoblè, Amoula, Dantèkewo*, Daouda*.
Alayomdè	Ouaké	11	10	Oroutani, Danwari, Ossoukpana, Tcholotcholo, Sambayé*, Kolor, Gnidou, Douroubayesirou, Singou, Agogo, Epkêtê.
Assaba	Bantè	14	12	Amoula, Inaimbo*, Eguede*, Dôdô, Effourou, Gangni, Okogan, Danwari, Kratchi, Laboko, Lassirin, Mondji, Oroutani, Morokorou.
Assotè	Ouaké	8	7	Gnidou, Heapala, Danwari, Agogo, Aliba*, Amoula, Kolor, Portchahabim.
Dendougou	Djougou	10	9	Morokorou, Naganganbinan*, Noukpam, Tabané, Baniouré, Noudorsi, Portchahabim, Zambê, Woroutani, Yonuan.
Fanbérékou	Péhonco	14	12	Ahimon, Mondji, Oroutani, Baniouré, Singou, Djatouba, Danwari, Tchée, Dôdô*, Douroubayessirou, Kpanhoura, Laboko, Wonnina*, Morokorou.
Gbéré	Ouoghi	10	10	Offêgui, Katala, Laboko, Djiladja, Effourou, Ossèmon, Tognibo, Inanwai, Obalè, Ahimon.
Koko	Tchaourou	16	11	Amoula, Wokiri, Kokorogbanou, Kokoro_kopargo*, Alo, Baniouré, Gnidou, Kokoro-koumakou, Kokoro kpédékpédé*, Kokoro-sencomou*, Kokouman, Gaboubaba*, Yakarango, Laboko, Lassirin, Saria*.
Fo-Bouko	Sinendé	17	12	Ahimon, Baniouré, Baniakpa, Banioure_pika*, Danwari, Douroubayésirou, Banioure_kpassikoba*, Kpanhoura, Laboko, Morokorou, Noulassi, Orouguiwa*, Oroutani, Soussouka, Bakpanatini*, Wossou*, Yaassi.
Frignon	Frignon	6	4	Amoula, Deyossira*, Gninnoubokokanmion, Danwari, Ossoukpana, Woroutani.
Orokoto	Glazoué	14	14	Laboko, Agatou, Ekpêtê, Morokorou, Kodjewe, Wété, Mafôbô, Effourou, Dôdô, Mondji, Kratchi, Gnalabo, Gangni, Okogan.
Ouoghi	Ouoghi	8	8	Laboko, Djilaja, Gangni, Effourou, Djatouba, Gnidou, Ouroutani, Taala.
Péporiakou	Toucountouna	8	2	Yaassi, Wonnifeenan*, Wossou*, Ounonyahoun*, Gakatele*, Feetani*, Soussouka, Itchankoe*.
Sakarou	N’dali	14	11	Baniakpa, Wankpa*, Ossousounou*, Kpounan, Baniouré, Oroutani, Danwari, Douroubayessirou, Kpanhoura, Laboko, Kinkerekou, Morokorou, Soussouka, Tampanou*.
Tallou	Frignon	5	3	Amoula, Sombanuanga*, Danwari, Oroutani*, Noukpam.
Tchêtti	Tchêtti	14	12	Ahimon, Ossemou*, Feetani*, Amoula, Dôdô, Effourou, Gangni, Gnidou, Okogan, Danwari, Kratchi, Laboko, Lassirin, Oroutani.
Tokotoko	Tokotoko	10	6	Tampanou*, Tinondaati, Laafoun*, Nanganganbinan*, Noudorsi, Portchahabim, Wouroutani, Yonuan, Damboura*, Amoula.
Tchakalakou	Natitingou	10	6	Nouanla_pangui*, Nouanla_poua*, Oroutani, Koumassi_nonbou*, Laboko, Sokodoï, Nouanla_siibi*, Soussouka, Taatimanin, Ossousounou.
Katabam	Djougou	7	5	Oroutani, Warmai, Tamsam, Yaassi, Adoro, Assina*, Baanon*.

NPLV: Number of popular yam landraces identified in the village, NPLM: Number of popular yam landraces identified in the market.

* Popular yam landraces not found in the markets surrounding the surveyed village.

In the markets, the price of tubers of the identified popular yam varied from one landrace to the other (Table 3). Forty-four popular white Guinea yam landraces which had a high price (≥ 350 CFA franc ≈ 0.62 $ per kg), were available in high quantity (≥ 1 tone), and sold by many sellers (≥ 5) in the markets (Table 3). The landrace Laboko was the most expensive (650 CFA franc ≈ 1.14 $ per kg) and Gnidou landrace was the cheapest (350 CFA franc/kg). Majority (72.7%) of these white Guinea yam landraces are early maturing with double harvest per year. Subject to synonymy, the Donga department had the highest number (31) of popular yam landraces with high market value, followed by the Collines (30), Borgou (22), and Atacora (18) departments (Table 3).

**Table 3:** Market value and distribution of the popular yam landraces in the study area.

Landraces	Cycle	Price per Kilogram (CFA franc)	Distribution per department			
			C	D	A	B
Adoro	Late maturity	450	**-**	**+**	**-**	**-**
Agatou	Early maturity	475	**+**	**+**	**-**	**-**
Agogo	Early maturity	525	**-**	**-**	**+**	**+**
Ahimon	Early maturity	600	**+**	**+**	**+**	**+**
Amoula	Early maturity	550	**+**	**+**	**+**	**+**
Baniakpa	Late maturity	550	**-**	**-**	**-**	**+**
Baniouré	Early maturity	550	**-**	**+**	**+**	**+**
Danwari	Early maturity	450	**+**	**+**	**+**	**+**
Déba	Late maturity	450	**+**	**+**	**-**	**+**
Djatouba	Early maturity	350	**+**	**+**	**-**	**-**
Djiladja	Early maturity	500	**+**	**-**	**-**	**-**
Dôdô	Early maturity	500	**+**	**-**	**+**	**+**
Douroubayésirou	Early maturity	450	**-**	**+**	**+**	**+**
Effourou	Early maturity	500	**+**	**-**	**-**	**-**
Gangni	Early maturity	450	**+**	**-**	**-**	**-**
Gnalabo	Early maturity	450	**+**	**+**	**-**	**-**
Gnidou	Early maturity	350	**+**	**+**	**-**	**-**
Kinkérékou	Late maturity	450	**+**	**+**	**+**	**+**
Kodjéwé	Early maturity	550	**+**	**-**	**-**	**-**
Kpanhoura	Late maturity	450	**-**	**-**	**-**	**+**
Kratchi	Early maturity	550	**+**	**+**	**-**	**-**
Laboko	Early maturity	650	**+**	**+**	**+**	**+**
Lassirin	Early maturity	450	**+**	**+**	**-**	**-**
Mondji	Early maturity	500	**+**	**+**	**-**	**-**
Morokorou	Early maturity	600	**+**	**+**	**+**	**+**
Noudorsi	Early maturity	500	**-**	**+**	**-**	**-**
Noukpam	Late maturity	500	**-**	**+**	**-**	**-**
Okogan	Early maturity	450	**+**	**-**	**-**	**-**
Oroutani	Early maturity	500	**+**	**+**	**+**	**+**
Ossoukpana	Early maturity	450	**+**	**+**	**+**	**+**
Portchahabim	Late maturity	450	**-**	**+**	**-**	**-**
Singou	Late maturity	450	**+**	**-**	**+**	**+**
Foutoukpete	Early maturity	400	**-**	**+**	**-**	**-**
Soussouka	Early maturity	500	**+**	**+**	**+**	**+**
Taatimanin	Early maturity	500	**-**	**-**	**+**	**-**
Tabané	Late maturity	450	**+**	**+**	**+**	**+**
Tamsam	Late maturity	400	**-**	**+**	**-**	**+**
Tchéé	Early maturity	400	**+**	**+**	**+**	**+**
Tcholotcholo	Early maturity	400	**-**	**+**	**-**	**-**
Tognibo	Early maturity	450	**+**	**-**	**-**	**-**
Warmai	Late maturity	450	**-**	**+**	**-**	**-**
Yakarango	Late maturity	450	**+**	**+**	**-**	**+**
Yaassi	Early maturity	500	**+**	**+**	**+**	**+**
Yoblè	Early maturity	450	**+**	**-**	**-**	**-**

C: Collines, D: Donga, A: Atacora, B: Borgou

### 3.2. Participatory evaluation

**Fig 1:**
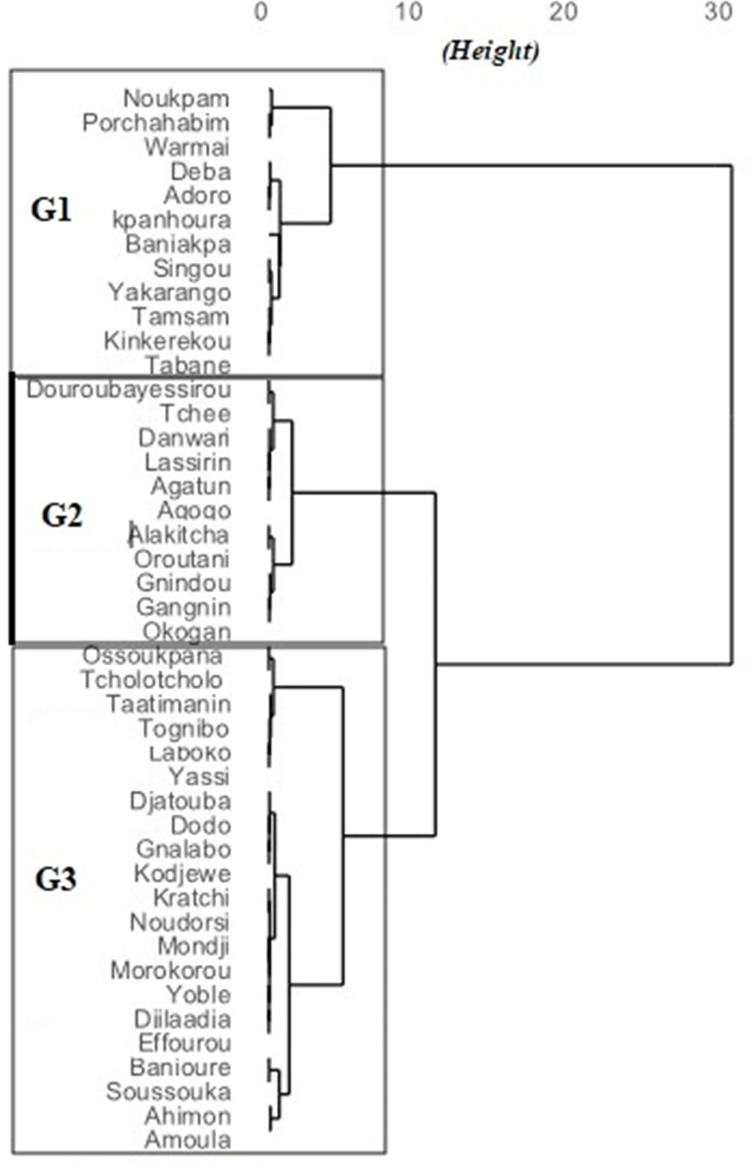
Dendrogram of popular yam landraces with high market value based on their agronomic, technological and culinary characteristics according to the surveyed farmers.

According to the surveyed farmers of the 44 popular white Guinea yam landraces with high market value recorded, 40 were perceived as possessing very good pounding ability, 34 were very good as boiled yam, 32 were very good as fried yam while 34 produced high quality yam chips (Table 4). Farmers perceived most of these white Guinea yam landraces as having relatively long post-harvest storage ability (32), high productivity (36), tolerance to weeds (35), tolerance to drought (20), and adaptation to poor soils (29). Some elite white Guinea yam landraces have been reported to be tolerant to anthracnose (25), yam mosaic virus (24), and nematodes (29). Using the hierarchical clustering method, the 44 popular yam landraces with high market value were classified into three groups (Fig 1). The first group included 12 late-maturating yam landraces (10 to 12 months to maturity), namely “Kokoro group of varieties” that have a single harvest and produce many small tubers, commonly used for the manufacture of yam chips. The second group included 11 early-maturing yam landraces (6 to 8 months to maturity) found to be highly susceptible to low soil moisture conditions, poor soil fertility, anthracnose disease, virus, and weeds. However, some landraces in this second group, namely Gnidou, Okagan and Agatou, were perceived by farmers as resistant to nematodes and having high productivity with very good quality as boiled yam. The twenty-one popular yam landraces of group 3 have early maturity are moderately productive, tolerant to high soil moisture but highly susceptible to nematodes, anthracnose and the yam mosaic virus.

**Table 4:** Farmers’ perceptions of agronomic, technological and culinary performance of popular yam landraces with high market value in the study area.

Evaluation traits	Modalities	Acceptability level (Number of landraces)	
		Very good	Acceptable
Culinary	Pounded yam	40	4
	Boiled yam	34	10
	Quality of fried yam	32	12
	Quality of yam chips	34	10
	Yam chips storability	32	12
Agronomic	Relative productivity	36	8
	Resistance to poor soil	29	15
	Resistance to drought	20	24
	Tolerance to soil moisture	26	18
	Flesh tuber storability	26	18
	Tolerance to weeds	35	9
Sanitary	Tolerance to nematode	29	15
	Tolerance to anthracnose	25	19
	Tolerance to YMV	24	20

### 3.3. Agronomic performance of yam landraces

#### 3.3.1. On-farm yield

**Fig 2:**
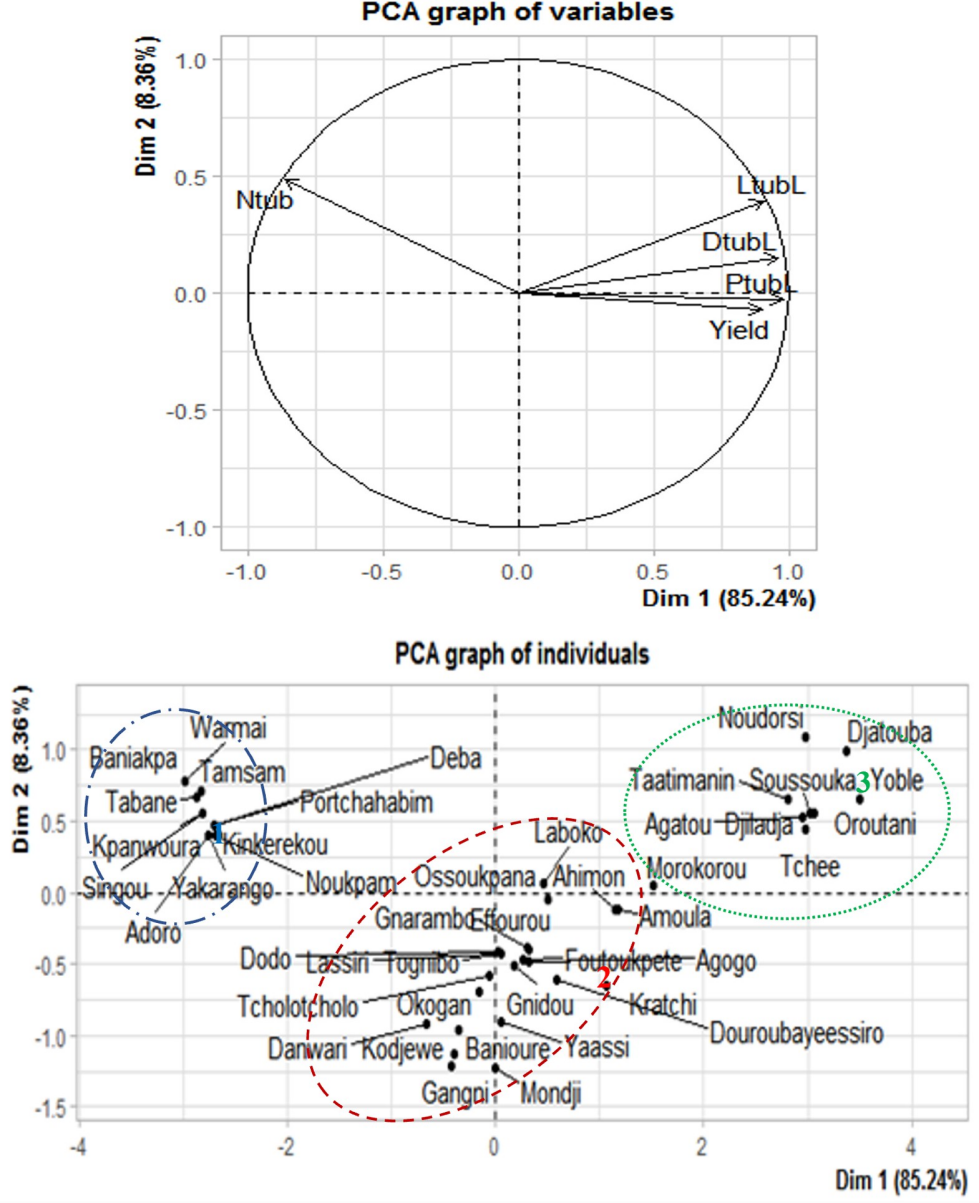
Correlation matrix between yield and its components and projection of popular white Guinea yam landraces with high market value in the system of the two first axes.

High coefficient of variability for yield (CV = 51.6%), number of harvested marketable tubers per mound (CV = 52.4%), marketable tuber weight per mound (CV = 38.7%), marketable tuber length (CV = 31.4%), and width (CV = 30.3%) were observed among the popular white Guinea yam landraces (Table 5). The yield of the popular white Guinea yam landraces ranged from 2.0 ± 0.5 kg/mound (Kpanhoura landrace) to 8.6 ± 0.8 kg/mound (Amoula landrace) with an average of 4.6 ± 1.8 kg/mound. The average yam tuber length and width were 39.4 ± 11.3 cm, and 28.6 ± 11.4 cm, respectively. The Déba landrace had significantly lower marketable tuber weights per mound (1.5 ± 0.2 kg) and higher number of harvested marketable tubers per mound (2.6 ± 0.9). Tognibo landrace had the smallest marketable tubers (Table 5). Results of correlation analysis revealed a positive and highly significant association between yield and marketable tuber weight (Fig 2). Similarly, tuber length, width, and diameter displayed positive correlations with the weight yield per plant (Fig 2). However, negative correlation was observed between the number of tubers per mound and the weight of the marketable tubers.

**Table 5:** Average yield, and yield components of the 44 popular yam landraces with high market value.

	Landraces	Yield (Kg/mound)			PtubL	Ntub	LtubL (cm)	DtubL (cm)
		Mean ± SD	Min	Max				
1	Adoro	3.9 ± 0.6bcdefghi	3.1	4.5	3.9 ± 0.6defghi	1.0 ± 0.0a	29.4 ± 1.9cd	14.2 ± 2.5bc
2	Agatou	4.6 ± 0.7cdefghi	3.8	5.5	4.4 ± 0.8efghij	1.2 ± 0.4a	32.7 ± 8.6cde	16.3 ± 3.7bcde
3	Agogo	5.6 ± 1.5defghi	3.7	7.6	5.6 ± 1.5ghij	1.0 ± 0.0a	36.3 ± 4.1cdef	35.4 ± 3.4fghijk
4	Ahimon	6.6 ± 0.6ghi	4.9	6.4	5.7 ± 1.7ghij	1.6 ± 0.9ab	35.5 ± 7.8cde	30.9 ± 5.2efghijk
5	Amoula	8.6 ± 0.8i	3.9	10	7.6 ± 2.2j	1.4 ± 0.8a	48.1 ± 15.6cdef	31.8 ± 6.8efghijk
6	Baniakpa	3.3 ± 0.9abcdefg	2.2	4.5	2.8 ± 1.3abcdef	1.6 ± 0.5ab	35.9 ± 1.7cdef	21.9 ± 10.9bcdefghij
7	Baniouré	4.0 ± 1.3bcdefghi	2.0	5.3	3.4 ± 1.6bcdefgh	1.6 ± 0.9ab	27.6 ± 6.1bc	17.9 ± 12.2bcd
8	Danwari	5.1 ± 1.3cdefghi	3.9	6.7	4.8 ± 1.1efghij	1.2 ± 0.4a	37.6 ± 9.3cdef	35.5 ± 10.2 fghijk
9	Déba	3.3 ± 0.9abcdefg	2.4	4.7	1.5 ± 0.2a	2.6 ± 0.9c	33.6 ± 9.2cde	20.5 ± 8.6bcdefghi
10	Djatouba	2.8 ± 0.5abcd	2.3	3.5	2.8 ± 0.5bcdefg	1.0 ± 0.0a	35.3 ± 3.4cdef	29.5 ± 1.0defghijk
11	Djiladja	4.9 ± 1.4cdefghi	3.3	7.0	4.9 ± 1.4efghij	1.0 ± 0.0a	39.2 ± 7.7cdef	17.7 ± 3.9bcdef
12	Dôdô	6.7 ± 0.8ghi	5.7	7.5	6.7 ± 0.8ij	1.0 ± 0.0a	59.2 ± 2.6ef	40.7 ± 1.6jk
13	Douroubayésirou	5.5 ± 1.9defghi	3.7	8.1	5.5 ± 1.9fghij	1.0 ± 0.0a	35.4 ± 8.5cde	28.5 ± 4.1cdefghijk
14	Effourou	3.5 ± 2.0abcdef	1.8	6.1	3.5 ± 2.0bcdefgh	1.0 ± 0.0a	43.5 ± 5.8cdef	33.2 ± 9.0efghijk
15	Foutoukpete	3.3 ± 0.6abcdefgh	2.7	4.2	3.3 ± 0.6bcdefghi	1.0 ± 0.0a	30.4 ± 2.8cd	29.5 ± 2.8defghijk
16	Gangni	5.7 ± 2.3defghi	1.7	7.3	5.7 ± 2.3fghij	1.0 ± 0.0a	49.5 ± 13.8cdef	37.7 ± 8.3hijk
17	Gnalabo	4.3 ± 0.5cdefghi	3.9	5.2	4.3 ± 0.5efghij	1.0 ± 0.0a	37.6 ± 4.4cdef	35.0 ± 5.6fghijk
18	Gnidou	4.8 ± 1.1cdefghi	3.3	6.0	4.8 ± 1.1efghij	1.0 ± 0.0a	42.5 ± 13.8cdef	25.0 ± 7.2bcdefghijk
19	Kinkérékou	4.7 ± 1.7cdefghi	3.6	7.8	4.7 ± 1.7efghij	1.0 ± 0.0a	33.3 ± 3.3cde	24.1 ± 1.0bcdefghijk
20	Kodjéwé	4.9 ± 1.4cdefghi	3.3	7.0	4.9 ±1.4efghij	1.0 ± 0.0a	39.2 ± 7.7cdef	17.7 ± 3.9bcdef
21	Kpanhoura	2.0 ± 0.5a	1.5	2.7	2.0 ± 0.5abc	1.0 ± 0.0a	32.2 ± 3.3cde	14.5 ± 1.7bc
22	Kratchi	3.2 ± 0.8abcdef	2.5	4.5	2.9 ± 0.9bcdefg	1.4 ± 0.5ab	35.5 ± 1.9cdef	18.3 ± 9.2bcdef
23	Laboko	2.9 ± 0.9abcde	1.7	4.1	1.8 ± 0.6ab	1.6 ± 0.5ab	41.0 ± 11.5cdef	32.0 ± 2.7efghijk
24	Lassirin	4.7 ± 2.0cdefghi	2.7	7.1	4.1 ± 1.6defghi	1.4 ± 0.5ab	40.9 ± 2.6cdef	37.5 ± 2.6hijk
25	Mondji	4.4 ± 1.3cdefghi	2.7	5.9	4.4 ± 1.3efghij	1.0 ± 0.0a	36.3 ± 7.2cdef	27.9 ± 9.7cdefghijk
26	Morokorou	5.7 ± 0.5efghi	5.1	6.2	5.7 ± 0.5ghij	1.0 ± 0.0a	44.9 ± 0.8cdef	42.5 ± 3.2k
27	Noudorsi	6.4 ± 2.4fghi	3.4	9.7	6.4 ± 2.4hij	1.0 ± 0.0a	44.5 ± 6.6cdef	42.1 ± 8.1jk
28	Noukpam	5.3 ± 0.9defghi	4.3	6.8	5.3 ± 0.9ghij	1.0 ± 0.0a	35.1 ± 7.7cde	25.0 ± 14.3bcdefghijk
29	Okogan	5.5 ± 0.7defghi	4.8	6.6	5.5 ± 0.7ghij	1.0 ± 0.0a	46.0 ± 4.0cdef	35.6 ± 2.1fghijk
30	Oroutani	3.6 ± 1.3abcdefgh	1.7	4.9	3.6 ± 1.3cdefghi	1.0 ± 0.0a	42.4 ± 3.2cdef	31.2 ± 5.6efghijk
31	Ossoukpana	5.7 ± 1.2defghi	4.3	7.0	5.7 ± 1.2ghij	1.0 ± 0.0a	42.6 ± 10.9cdef	13.2 ± 4.9b
32	Portchahabim	2.1 ± 0.4ab	1.7	2.8	2.1 ± 0.4abcd	1.0 ± 0.0a	39.8 ± 1.3cdef	26.8 ± 1.8cdefghijk
33	Singou	3.0 ± 0.7abcdef	2.1	3.7	3.0 ± 0.7bcdefgh	1.0 ± 0.0a	38.1 ± 5.1cdef	31.7 ± 1.4ghijk
34	Soussouka	2.6 ± 0.8abc	1.3	3.3	2.6 ± 0.8abcde	1.0 ± 0.0a	43.4 ± 3.8cdef	32.5 ± 5.8efghijk
35	Taatimanin	6.0 ± 2.0defghi	2.8	7.8	6.0 ± 2.0ghij	1.0 ± 0.0a	53.9 ± 3.8def	45.7 ± 10.7k
36	Tabané	3.2 ± 1.7abcdef	1.9	6.1	2.7 ± 1.4abcde	2.0 ± 0.7b	20.3 ± 4.6b	17.3 ± 3.2bcdef
37	Tamsam	5.9 ± 1.9defghi	3.5	7.8	5.9 ± 1.9ghij	1.0 ± 0.0a	46.9 ± 14.8	19.2 ± 5.7bcdefgh
38	Tchéé	6.9 ± 1.1hi	5.8	8.4	6.9 ± 1.1ij	1.0 ± 0.0a	64.4 ± 2.8f	34.5 ± 16.5efghijk
39	Tcholotcholo	4.0 ± 1.1bcdefghi	2.8	5.5	4.0 ± 1.1defghi	1.0 ± 0.0a	32.7 ± 3.4cde	17.7 ± 2.6bcdefg
40	Tognibo	4.4 ± 1.1cdefghi	3.5	6.3	4.4 ± 1.1efghij	1.0 ± 0.0a	13.2 ± 20.7a	11.4 ± 16.6a
41	Warmai	5.5 ± 0.6defghi	2.8	4.1	5.5 ± 0.6ghij	1.0 ± 0.0a	51.8 ± 2.3def	38.4 ± 6.6hijk
42	Yaassi	3.8 ± 0.7abcdefghi	2.5	4.3	3.8 ± 0.7cdefghi	1.0 ± 0.0a	36.5 ± 5.3cdef	33.9 ± 6.9efghijk
43	Yakarango	6.2 ± 2.6efghi	3.4	9.7	6.2 ± 2.6ghij	1.0 ± 0.0a	43.3 ± 9.5cdef	40.5 ± 8.6ijk
44	Yoblè	5.7 ± 0.5efghi	5.1	6.2	5.7 ± 0.5ghij	1.0 ± 0.0a	44.9 ± 0.8cdef	42.5 ± 3.2k
	**Total mean**	**4.6 ± 1.8**	**1.3**	**10**	**4.5 ± 1.9**	**1.2 ± 0.4**	**39.4 ± 11.3**	**28.6 ± 11.4**

SD: Standard deviation; PtubL: Weight of marketable tubers per mound; Ntub: Number of marketable tubers harvested per mound; LtubL: Marketable tuber length; DtubL: Marketable tuber width.

Results of the principal component analysis showed that the first axis explained 85.24% of the total variability. The length (LtubL), diameter (DtubL), and weight (PtubL) of tubers and yields were found to be positively correlated with the first axis, while the number of harvested tubers per mound (Ntub) was negatively correlated with the first axis (Fig 2). The principal component analysis clustered the popular white Guinea yam landraces with high market value into three groups with the 12 yam landraces in the first group producing a relatively higher number of tubers per mound. The second group comprising 23 yam landraces had moderate yield performance. The third group comprised nine yam landraces with higher yield as well as large and long yam tubers.

#### 3.3.2. Flowering and fructification

Twenty-nine elite yam landraces with high market values had low flowering capacity. Of these yam landraces, 24 yam landraces had flowering capacity but no fruit production, whereas five landraces were found to be completely sterile (Table 6). Two popular yam landraces (Dodo and Effourou) exhibited half flowering and fructification capacities. Seven of the popular yam landraces had high flowering capacity (Table 6). However, only Danwari, Kodjewe, Mondji, and Gnidou landraces had very good potential for flowering and fruit production.

**Table 6:** Flowering capacity and fructification of the 44 popular yam landraces with high market value recorded in the study area.

Parameters	Variables	Number	Landraces
**Flowering**	No flowers	5	Lassiri, Singou, Tabane, Tamsam, Warmai
	Low	29	Djatouba, Djiladja, Laboko, Noudorsi, Oroutani, Ossoukpana, Taatimanin, Tchee, Yakarango, Ahimon, Adoro, Foutoukpete, Kinkerekou, Okogan, Tcholotcholo, Agatou, Douroubayeessiro, Kpanwoura, Kratchi, Morokorou, Noukpam, Yoble, Agogo, Amoula, Banioure, Gangni, Soussouka, Tognibo, Gnarambo
	Medium	3	Baniakpa, Dodo, Effourou
	High	7	Deba, Portchahabim, Yaassi, Gnidou, Danwari, Mondji, Kodjewe
**Fructification**	No Fruit	24	Lassiri, Singou, Tabane, Tamsam, Warmai, Djatouba, Djiladja, Laboko, Noudorsi, Oroutani, Ossoukpana, Taatimanin, Tchee, Yakarango, Ahimon, Adoro, Deba, Foutoukpete, Kinkerekou, Okogan, Tcholotcholo, Baniakpa, Portchahabim, Yaassi
	Low	14	Agatou, Douroubayeessiro, Yoble, Kpanwoura, Kratchi, Morokoro, Noukpam, Agogo, Amoula, Banioure, Gangni, Soussouka, Tognibo, Gnarambo
	Medium	2	Dodo, Effourou
	High	4	Danwari, Kodjewe, Mondji, Gnidou

#### 3.3.3. Phytosanitary status

Under on-farm conditions, several of the 44 popular yam landraces were found to be susceptible to anthracnose, virus and nematodes and had relatively high incidence of infections, ranging from 10 to 92%, 10 to 100% and 0 to 66%, respectively (Table 7). Very few yam landraces were found to be tolerant to the YMV disease. Only Djiladja (Incidence (Inc) = 10, mean severity (Ms) = 1.1), and Ossoukpana (Inc = 10, Ms = 1.5) landraces resisted the YMV infestation. Amoula (Inc = 10, Ms = 1.33), and Porchahabim (Inc = 10, Ms = 1.8) landraces were tolerant to the anthracnose disease. Adoro (Inc = 0, Ms = 1), Agatou (Inc = 0, Ms = 1), Djiladja (Inc = 0, Ms = 1), Gnidou (Inc = 0, Ms = 1), Kratchi (Inc = 0, Ms = 1), Okogan (Inc = 0, Ms = 1), Tchéé (Inc = 0, Ms = 1) and Yoblè (Inc = 30, Ms = 1.6) were reported to be nematode resistant and had low foliar severity of *S*. *bradys* infestation.

**Table 7:** Phytosanitary status of popular varieties of yam from Benin.

Landraces	YMV		ANTHRACNOSE		NEMATODES	
	Inc	Ms	Inc	Ms	Inc	Ms
Adoro	70	2.0	90	2.2	0	1.0
Agatou	50	1.5	50	1.5	0	1.0
Agogo	50	1.3	50	1.5	50	1.5
Ahimon	90	1.5	40	1.4	50	2.0
Alakitcha	50	1.5	50	1.5	0	1.0
Amoula	50	1.5	10	1.33	50	1.5
Baniakpa	70	1.9	33.3	1.3	50	1.7
Baniouré	50	1.8	40	1.4	50	1.7
Danwari	50	1.5	50	1.5	50	1.5
Deba	60	2.1	50	2.0	50	1.5
Djatouba	50	1.9	50	1.8	50	1.5
Djiladja	10	1.1	30	1.3	0	1.0
Dôdô	80	2.3	50	2.0	50	1.5
Douroubayessirou	70	2.0	80	1.8	50	1.5
Effourou	60	1.6	80	1.9	50	1.5
Gangnin	50	1.6	50	1.3	50	1.7
Gnalabo	66.7	2.0	40	1.4	50	1.5
Gnindou	100	2.2	60	1.9	0	1.0
Kinkerekou	66.7	1.9	40	1.3	50	1.3
Kodjèwé	60	1.8	50	1.4	50	1.7
Kpanhoura	60	1.9	48	1.5	50	2.0
Kratchi	54.7	2.0	48	1.4	0	1.0
Laboko	90	2.0	50	1.9	50	2.3
Lassirin	50	2.0	52	1.3	30	1.7
Mondji	60	2.0	54	1.3	50	1.7
Morokorou	70	2.1	56	1.4	50	1.5
Noudorsi	50	2.1	58	1.5	50	1.4
Noukpam	50	2.1	60	2.0	40	1.5
Okogan	60	2.1	62	1.8	0	1.0
Oroutani	50	1.8	40	1.3	50	1.5
Ossoukpana	10	1.5	50	2.0	50	1.5
Porchahabim	80	2.1	10	1.8	50	2.0
Singou	80	1.1	33.3	1.1	59	1.6
Soussouka	70	1.1	40	1.3	66	1.5
Taatimanin	60	2.3	50	1.4	50	1.5
Tabané	50	2.0	50	1.9	50	1.3
Tamsam	66.7	2.3	50	1.3	40	1.7
Tchéé	56.7	2.3	30	1.4	0	1.0
Tcholotcholo	54.7	2.3	82	1.9	50	1.5
Tognibo	52.6	2.3	84	1.3	40	1.5
Warmai	50.6	2.4	86	1.4	45	1.6
Yakarango	48.6	2.4	88	1.5	20	2.0
Yassi	46.6	2.4	30	1.4	40	2.0
Yoblè	44.6	2.4	92	1.5	30	1.6

Inc: Incidence; Ms: Mean severity; YMV: Yam mosaic virus

### 3.4. Sensory evaluation

Majority (81.8%) of popular yam landraces with high market values were preferred in both boiled and pounded forms. Only 6.8% and 11.4% landraces were preferred in the boiled and pounded forms, respectively. Ahimon, Baniouré, Déba, Douroubayésirou, and Kratchi landraces were most preferred in the pounded form while, Adoro, Ossoukpana, and Singou landraces were the most preferred in the boiled form. The hedonic tests of boiled and pounded yam of the 44 popular white Guinean landraces with high market value showed that more than half of them were classified as pleasant (95.6%), and 4.5% as very pleasant by the tasters. Boiled and pounded form of Laboko and Djiladja landraces were considered as very pleasant by all the taste panellists. A significant variability in the scores obtained for pounded tubers of the different yam landraces was observed in terms of colour (CV = 29.8%), sweetness (CV = 33.1%), hardness (CV = 34.6%), aroma (CV = 168.1%), presence of lumps (CV = 199.1%) and elasticity (CV = 30.6%). The sweetness of Laboko and Tchee landraces were most appreciated by the panellists, while Tamsam and Baniouré landraces were the least appreciated for this attribute. Okogan, Tamsam, and Gnarambo landraces were considered by panellists as producing pounded yam with good texture attributes. In terms of the elasticity of pounded yam, Laboko had the highest score by the panellists (Table 8). Deba, Djiladja, Laboko, and Tabane landraces were adjudged by the panellists as having the most attractive aroma. The unpleasant presence of lumps in pounded yam, attracted a high score (1) in the Douroubayésirou, Effourou, Tchée, and Tognibo landraces.

**Table 8:** Descriptive statistics of the scores of the studied parameters of the landraces.

Landraces	Mean score for attributes						
	Time (min)	Colour	Sweetness	Aroma	Hardness	Lumps	Elasticity
Adoro	23	1	2.5	0	3	0	1.4
Agatou	19	1.6	1.5	0.1	2.6	0	3.2
Agogo	24	1.3	2.5	0	2	0	2.1
Ahimon	27	1.9	2.5	0	2	0	1.9
Amoula	22	0.8	2.1	0.1	1.7	0	2.8
Baniakpa	28	1.3	1	0	1.8	0	2.2
Banioure	25	1	0.8	0	2	0	2.6
Danwari	20	1	1.9	0	2.	0	3
Deba	18	2	2	1	1.5	0	1
Djatouba	19	1.6	1.5	0.1	2.6	0	3.2
Djiladja	31	0.9	2.9	1	1.6	0	2.5
Dodo	17	1.3	2.1	0.1	1.9	0	2.9
Douroubayésirou	26	1	2.1	0.2	2.8	1	2.4
Effourou	14	1.7	1.1	0	2.3	1	2.8
Foutoukpete	24	1.3	2.5	0	2	0	2.1
Gangni	21	1	2.1	0	1.8	0	2.4
Gnarambo	35	1.3	1.7	0	4	0.8	2
Gnidou	16	1	1.8	0	2.7	0	1.3
Kinkerekou	22	0.9	1.3	0	2.3	0.4	1.8
Kodjewe	22	0.8	2.2	0.1	1.6	0.4	3.3
Kpanwoura	30	1	2	0.9	2	0	2.3
Kratchi	25	1.8	2.1	0	2.8	0.1	2.9
Laboko	18	1.9	4	1	1.5	0	3.6
Lassiri	15	2	1.2	0	1.5	0	1.2
Mondji	23	1	1.8	0	2.8	0.1	2.4
Morokorou	19	1	2.1	0	1.9	0	2.3
Noudorsi	10	1	1.5	0	1.9	0	1.4
Noukpam	20	1.	2.3	0.7	2	0.1	3.4
Okogan	35	1	1.3	0.1	4.2	0.8	3.4
Oroutani	24	1	2.1	0.2	2.8	0.1	3.4
Ossoukpana	20	1.9	2.3	0	2.2	0	2.4
Portchahabim	21	1	1.8	0	2.7	0	1.3
Singou	30	0.9	2	0	1.3	0	2.8
Soussouka	21	1	2	0.2	1	0	1
Taatimanin	34	1	2	0	3.3	0	2.8
Tabane	25	1.1	3	1	2.3	0	2.7
Tamsam	33	1.2	0.8	0.4	4.1	0	2.3
Tchee	31	0.9	3.7	0.8	3.8	1	1.9
Tcholotcholo	32	1.1	1.8	0	2.8	0.3	2.3
Tognibo	37	1	1.5	0.1	3.5	1	2.9
Warmai	27	0.7	1.7	0	4	0.1	2
Yaassi	10	1	2.1	0.3	1.2	0	1.3
Yakarango	18	1	1.3	0	2.2	0	1.3
Yoble	20	1.2	1.8	0.3	1.7	0	2.3

### 3.5. Cluster analysis

#### 3.5.1. Contribution of each category of variables to the explanation of the variations between yam landraces

**Fig 3:**
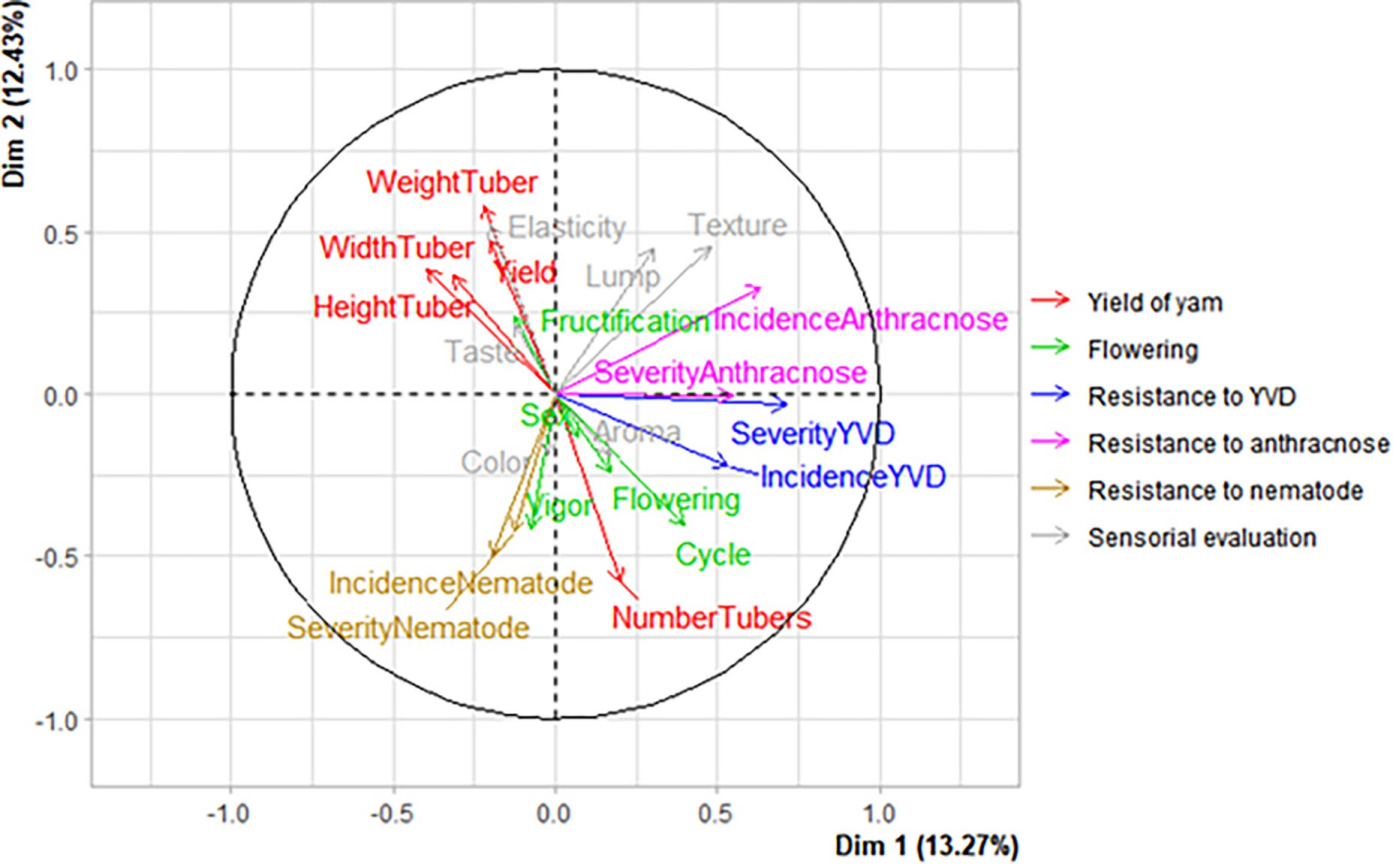
Percent contribution of each group of variables to the first Dimension (Dim 1) and second Dimension (Dim 2) factors derived from the multiple factor analysis performed on the characteristics of the yam landraces.

The multiple factor analysis showed that the first factor (Dim 1) explained 13.3% of the total variation among the yam landraces, the second factor (Dim 2) 12.4% while the first two axis explained 25.7% of the total variation among the yam landraces (Fig 3). The percent contribution to the inertia of the first factor was highest for resistance to yam viral infection and resistance to anthracnose traits (Fig 3). However, the percent contribution to the inertia of the second factor was highest for the sensorial character followed by the yam yield components, resistance to nematodes and the flowering characters.

#### 3.5.2. Classification and characteristics of popular yam landraces

**Fig 4:**
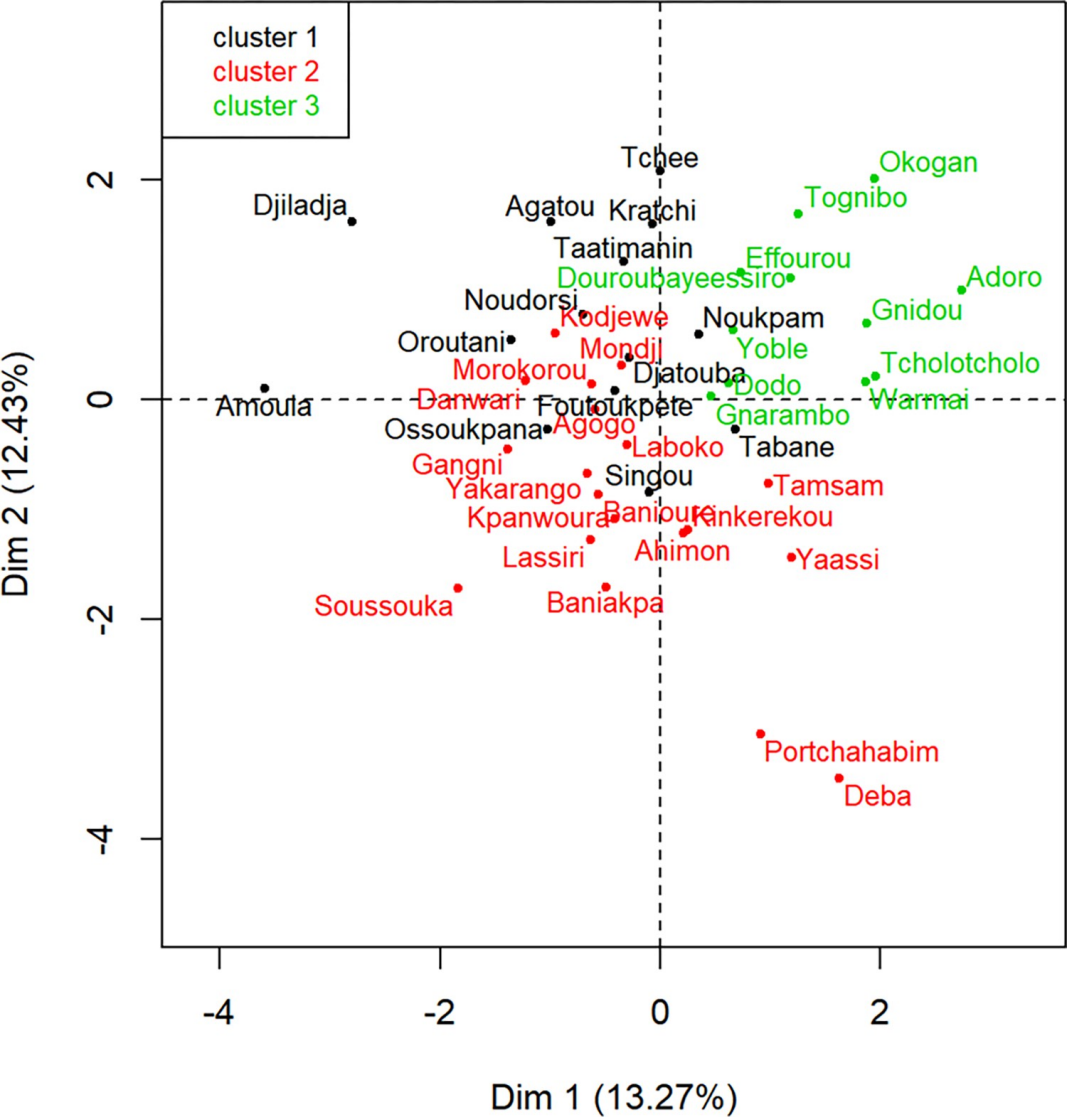
Position of yam landraces on the two first component (Dim 1 and 2) derived from the multiple factor analysis.

Fig 4 showed the projection of yam landraces on the first and second axes (Dim 1 and 2) derived from the multiple factor analysis. Table 9 showed the mean values for tuber yield and yield components and the mean scores of the qualitative variables for popular yam landraces within clusters derived from the hierarchical cluster analysis. The cluster 1 included yam landraces with high yield (4.9 kg/mound), big tubers with height of about 43.3 cm and weight of about 4.7 kg and width of about 31.4 cm as well as good taste and good elasticity of the pounded yam. Additionally, the cluster 1 yams exhibited tolerance to pests and diseases (low incidence of viral infection and anthracnose, and low severity of viral infection and nematodes). The cluster 2 included yam landraces characterized by high number of tubers (1.2), good plant vigour, and low incidence of anthracnose, but had the smallest tuber sizes, low mean yield (3.8 kg/mound), high incidence and severity of nematodes, low elasticity, and lump of pounded yam. The cluster 3 included yam landraces characterized by the highest incidence of anthracnose, severity of anthracnose and viral infection, and lump of pounded yam.

**Table 9:** Mean values of yield and yield components, and median values from scores of qualitative variables of popular yam landraces within clusters derived from the hierarchical cluster analysis (median values are in the square brackets).

Variables	Overall (n = 44)	Cluster1 (n = 14)	Cluster2 (n = 11)	Cluster3 (n = 19)
Quantitative traits	Mean [Median]			
Weight of tubers	3.8 [3.7]	4.7*[4.6]	3.6*[3.8]	3.4 [3.6]
Number of tubers	1.3 [1.2]	1.1 [1.1]	1.2*[1.2]	1.5 [1.2]
Yield	4.2 [4.0]	4.9*[5.1]	3.9*[4.0]	3.8 [3.7]
Height of tubers	36.3 [34.9]	43.3*[41.1]	32.5*[32.7]	33.4 [32.2]
Width of tubers	26.2 [25.9]	31.4*[33.3]	23.6 [25.1]	24.1 [23.7]

* v-test value > 1.96 representing statistically significant (p-value <0.05) difference between cluster mean and overall mean; or significant (p-value <0.05) difference between cluster median score and overall median score using Mann-Whitney test with 2 independent samples.

## 4. Discussion

Our results showed that, subject to local yam landraces only 44 of the 116 popular white Guinea yam landraces recorded in the study area had a high market value. Yam production in the study area is mainly intended for family consumption [35]. Consequently, farmers cultivate several yam landraces of low market value with the major goal of meeting their varietal preference criteria. Majority of the popular yam landraces with high market value recorded have early maturity and are known for their large tubers. Indeed, Banson and Danso [36] have shown that the price per kilogram of yam increased with the size of the tuber. The majority of the late yam landraces identified with high market value were mainly found in Donga department, which is the preferred area for producing yam chips for which these landraces are known for [23].

In general, farmers perceived most of the identified popular yam landraces with high market value as having high agronomic and culinary performances. Indeed, according to Zannou et al. [13], the price of different yam landraces in the market reflected their technological or taste characteristics. In addition, previous studies reported that, in general, yam landraces grown on a large scale by many households in a region tended to have better agronomic performance [8, 23]. Knowing that, in many cases synonymy exists among cultivated yam landraces in the Republic of Benin [3, 37, 38], the identified duplicates among this popular yam landraces with high market values by Agre et al. [39] is useful for their better conservation and use.

Our results showed that, the yield of the popular yam landraces with high market value varied significantly from one mound to another. This variation of yam yield could be due to an important interaction between the environment and the expression of their phenotypic potential [40]. In addition, it is known that yam yield is highly sensitive to climatic and pedological parameters such as precipitation, temperature, light, photoperiod and soil type [41]. Indeed, the rate of accumulation of photosynthetic reserves depends not only on the available vegetative mass [42], but also on geophysical conditions [40]. Multi-local tests should be carried out to identify growing-areas with optimal production conditions for each recorded landraces to boost yam yields in Republic of Benin.

Most of popular yam landraces with high market value recorded in this study showed poor flowering and fructification capacities. Indeed, it is known that natural pollination of white Guinea yam occurs very rarely in the field [43, 44], and this species has a very poor potential for flowering and fructification [45]. Landraces such as Danwari, Kodjewe, Mondji, and Gnidou, which presented a high flowering intensity with high fructification capacity, could be used in further yam genetic improvement.

Our results have revealed that the identified popular yam landraces with high market value in the Republic of Benin offer enormous potential for yam breeding programs. In fact, the recorded yam landraces that showed tolerance to diseases and nematodes, and had good agronomic and culinary characteristics should be prioritized for demonstration and use in yam breeding programs [1, 46]. Laboko and Djiladja landraces, which have organoleptic characteristics that satisfy consumer preferences could be used as outstanding varieties in yam breeding programs. In fact, Fakorede et al. [20] identified these landraces as satisfying the preferences of Beninese consumers and processors and are among the best parents for white Guinea yam breeding programs.

Our results suggested that a minimum yield of 4.16 ± 0.15 kg/mound, and tubers of minimum length and width of 36.41 ± 1.22 cm, and 25.44 ± 1.16 cm respectively must be the minimum standards that an improved variety of yam must have to be accepted by Beninese farmers. Furthermore, in addition to resistance to various biotic and abiotic stresses the improved variety should reveal minimum scores of 3.16 for texture, 0.75 for softness, 3.75 for elasticity, and 1.34 for colour preference during sensory evaluation to be acceptable by consumers. In the framework of a varietal improvement program for early maturity yam landraces, the MFA showed that landraces in cluster 1 would be good parents while the white Guinea yam landraces in cluster 2 would be ideal parents for a late-maturing yam for a breeding program.

## 5. Conclusions

Our study identified 44 popular white Guinea yam landraces with high market value and agronomic and culinary performance that could be very useful for market-oriented breeding programs. Among them, Danwari, Kodjewe, Mondji, and Gnidou landraces should be integrated in the national breeding programs because of their high flowering intensity, and fruit setting capacity. Amoula, Laboko, and Djilaadja landraces should be considered the standard landraces for high yield, good sensory attributes, and resistance to diseases and nematodes, respectively. This study allowed to identify basic breeding standards, which used by breeders could increase the acceptability of improved white Guinea yam varieties by farmers and consumers in the Republic of Benin.

## Supporting information

S1 Table This is the list of the surveyed villages and the corresponding sociolinguistic groups. (DOCX)
