# Supplementary material for: White Guinea yam (Dioscorea rotundata Poir.) landraces trait profiling and setting benchmark for breeding programs in the Republic of Benin
Source: PLoS One. 2022 Aug 17;17(8):e0273043. doi: 10.1371/journal.pone.0273043 (PMC9384998; doi:10.1371/journal.pone.0273043)
Supplement: S1 Table — (DOCX) [file pone.0273043.s001.docx]

| **Surveyed village** | **Surveyed market** | **Department** | **Sociolinguistic groups** |
| --- | --- | --- | --- |
| Glazoué | Glazoué | Collines | Idaatcha |
| Ouoghi | Savè | Collines | Tchabè |
| Koko | Tchaorou | Borgou | Tchabè |
| Gbébé | Savè | Collines | Tchabè |
| Adidokparou | Tchaorou | Borgou | Bariba |
| Sakarou | N'dali | Borgou | Bariba |
| Fo-Bouko | Sinendé | Borgou | Bariba |
| Fanbérékou | Péhunco | Borgou | Bariba |
| Tchakalakou | Toukountouna | Atacora | Wama |
| Tokotoko | Djougou | Donga | Yom |
| Assodè | Ouaké | Donga | Lokpa |
| Alayondè | Ouaké | Donga | Lokpa |
| Akaradè | Bassila | Donga | Kotocoli |
| Tallou | Frignon | Bassila | Lokpa |
| Frignon | Frignon | Donga | Ani |
| Assaba | Bantè | Collines | Nago-Fè |
| Tchètti | Tchètti | Collines | Nago-Fè |
| Katabam | Djougou | Donga | Yom |
| Péporiakou | Natitingou | Atacora | Wama |
| Dendougou | Djougou | Donga | Yom |
